# Simultaneous CSM‐TACE with CalliSpheres^®^ and partial splenic embolization using 8spheres^®^ for hepatocellular carcinoma with hypersplenism: Early prospective multicenter clinical outcome

**DOI:** 10.3389/fonc.2022.998500

**Published:** 2022-12-01

**Authors:** Jun Zhou, Zhuo Feng, Song Liu, Xiang Li, Ying Liu, Fei Gao, Jing Shen, Yue Wei Zhang, Guang Sheng Zhao, Ming Zhang

**Affiliations:** ^1^ Department of Radiology, The First Affiliated Hospital of Xi’an Jiaotong University, Xi’an, Shaanxi, China; ^2^ Minimally Invasive Interventional Diagnosis and Treatment Center, Affiliated Zhongshan Hospital of Dalian University, Dalian, Liaoning, China; ^3^ Department of Emergency, The First Affiliated Hospital of Dalian Medical University, Dalian, Liaoning, China; ^4^ Cancer Interventional Center, Linyi Cancer Hospital, Linyi, Shandong, China; ^5^ Hepatobiliary and Pancreatic Center, Beijing Tsinghua Changgung Hospital, Beijing, China; ^6^ Cancer Interventional Center, The Second Affiliated Hospital of Dalian Medical University, Dalian, Liaoning, China; ^7^ Department of Radiology, Affiliated Zhongshan Hospital of Dalian University, Dalian, Liaoning, China

**Keywords:** hepatocellular carcinoma, hypersplenism, partial splenic embolization, calliSpheres^®^, 8spheres^®^, CSM-TACE

## Abstract

**Background:**

Primary hepatocellular carcinoma is often complicated with hepatitis and liver cirrhosis. Some patients develop different degrees of splenomegaly, hypersplenism and hypohepatia due to the aggravation of liver cirrhosis, which to some extent interfere with the treatment of tumors and even affect the prognosis of patients. In this study, we prospectively evaluate the efficacy and safety of simultaneous CalliSpheres^®^ microspheres transcatheter arterial chemoembolization (CSM-TACE) and partial splenic embolization (PSE) using 8spheres^®^ for hepatocellular carcinoma (HCC) with hypersplenism.

**Methods:**

Ninety consecutive HCC patients with hypersplenism who underwent CSM-TACE were selected: 32 patients in CSM-TACE+PSE group, and 58 patients in CSM-TACE group. The peripheral blood cell counts (leukocyte, platelet (PLT), liver function and red blood cell (RBC)), CSM-TACE and/or PSE related complications, and the tumor control rate at 1 month after CSM-TACE were compared. The survival time and prognostic factors were also observed.

**Results:**

Before CSM-TACE, there were no significant differences in sex, age, Child-Pugh grade, tumor size, and alpha-fetoprotein (AFP) between the two groups. After CSM-TACE, the PLT and white blood cell (WBC) counts in CSM-TACE+PSE group were significantly higher than those in the CSM-TACE group (P<0.05). There were no significant differences in RBC before and after treatment (P > 0.05). In the CSM-TACE group, there were no significant differences in WBC, PLT, and RBC before and after treatment (P > 0.05). There was no significant difference in liver function at 1 month after treatment between the two groups. The cholinesterase (CHE) level in the CSM-TACE+PSE group after CSM-TACE+PSE was obviously higher than that before CSM-TACE+PSE and higher than that in the CSM-TACE group (P<0.05). However, the level of CHE returned to the preoperative level 1 month after CSM-TACE in the CSM-TACE group. The objective response rate (ORR) and median overall survival (OS) in the CSM-TACE+PSE group were higher than those in the CSM-TACE group (P<0.05). The adverse reactions of the two groups were fever, abdominal pain, stomach discomfort, nausea, and vomiting, and no serious complications occurred. The degree of abdominal pain and fever in the experimental group was lower than that in the control group (P > 0.05).

**Conclusions:**

Simultaneous CSM-TACE and PSE using domestic embolization particles for HCC with hypersplenism have good safety and efficacy and has a low incidence of PSE-related adverse events, it is conducive to improving liver function reserve, and can further improve the median OS.

## 1 Introduction

China is a country high prevalence of hepatitis B, and most hepatocellular carcinoma (HCC) patients in China have a background of post-hepatitis cirrhosis. Among them, more than 80% of HCC patients are accompanied by hypersplenism caused by liver cirrhosis (1). Hypersplenism not only leads to decreased levels of white blood cells and platelets, decreased appetite and digestive function, and impaired immune function, but also reduces blood flow back to the liver, thereby further promoting the occurrence of liver failure. Hyperactivity is an important factor in the poor prognosis of HCC (2–4). Partial splenic embolization is an effective therapeutic modality for the treatment of hypersplenism secondary to chronic liver disease. It is a simple, rapid procedure that is easily performed under local anesthesia; and it allows preservation of adequate splenic tissue to safeguard against overwhelming infection (5). In the field of liver transplantation, simultaneous partial splenectomy and liver transplantation (PSLT) is an effective treatment and should be performed in patients with advanced cirrhosis combined with severe splenomegaly and hypersplenism to prevent postoperative persistent hypersplenism (6). Moreover, both splenectomy and partial splenic embolization (PSE) were associated with the prevention of secondary sarcopenia in patients with liver cirrhosis (LC) (7). Therefore, PSE has gradually replaced splenectomy, and in patients with blunt splenic injury after splenic artery embolization, thromboembolism following splenic artery embolization (SAE) should be considered and thrombotic prophylaxis should be recommended (8).

Studies have confirmed that HCC control combined with simultaneous splenectomy has a good safety profile and can improve long-term survival (2, 3). Radiofrequency ablation or conventional transcatheter arterial chemoembolization (c-TACE) with simultaneous PSE in the treatment of patients with HCC and hypersplenism also has a good safety profile. It can not only increase the platelets and white blood cell levels, but also improve the liver reserve function of patients with HCC (4, 9), and can also improve the progression-free survival (PFS) and overall survival (OS) of patients (10). Up to now, there has been no report on the safety and efficacy of the application of drug-eluting beads TACE (DEB-TACE) simultaneous PSE in the treatment of HCC complicated with hypersplenism. Our preliminary research results suggest that the application of domestic CalliSpheres^®^ microspheres for DEB-TACE in the treatment of HCC has a good tumor control rate (11, 12), and it has achieved similar clinical effects with imported microspheres (12). CalliSpheres^®^ microspheres transcatheter arterial chemoembolization (CSM-TACE) has achieved good clinical efficacy in the treatment of unresectable advanced HCC, based on this, the purpose of this study is to observe the safety and efficacy of CSM-TACE simultaneous PSE using domestic 8spheres^®^ in patients with HCC and hypersplenism, thus provide a clinical basis for the feasibility of using domestic particulate embolic agents in the treatment of these HCC patients.

## 2 Materials and methods

### 2.1 General information

A total of 90 HCC patients who met the inclusion criteria were selected from January 2019 to January 2021 from 5 tumor intervention centers, including Affiliated Zhongshan Hospital of Dalian Univertity, Beijing Tsinghua Changgung Hospital, the Second Affiliated Hospital of Dalian Medical University, the First Affiliated Hospital of Dalian Medical University, and Linyi Cancer Hospital were eligible (**Table 1**).

**Table 1:** Baseline characteristics of the included objects and the target lesions.

Items	CSM-TACE+PSE (N=32)	CSM-TACE (N=58)	P
Gender			0.687
Male	30	53	
Female	2	5	
Age (yr)			0.482
>60	14	21	
≤60	18	37	
Alpha-fetoprotein (AFP) (ng/ml)			0.668
>400	17	33	
≤400	15	25	
Hepatitis			0.904
Yes	29	53	
No	3	5	
Child-Pugh			0.378
A	24	48	
B	8	10	
PVTT			0.813
Yes	5	8	
No	27	50	
Diameter (cm)			0.848
≤5	10	17	
>5	22	41	
Tumor number			
Solitary	9	18	
Multiple	23	40	
BCLC			0.928
B	19	35	
C	13	23	
Hypersplenism			0.967
Mild	6	12	
Moderate	20	36	
Severe	6	10	
Hemocytopenia			<0.001
One type	3	34	
Two types	20	24	
Three types	9	0	

**Figure 1:**
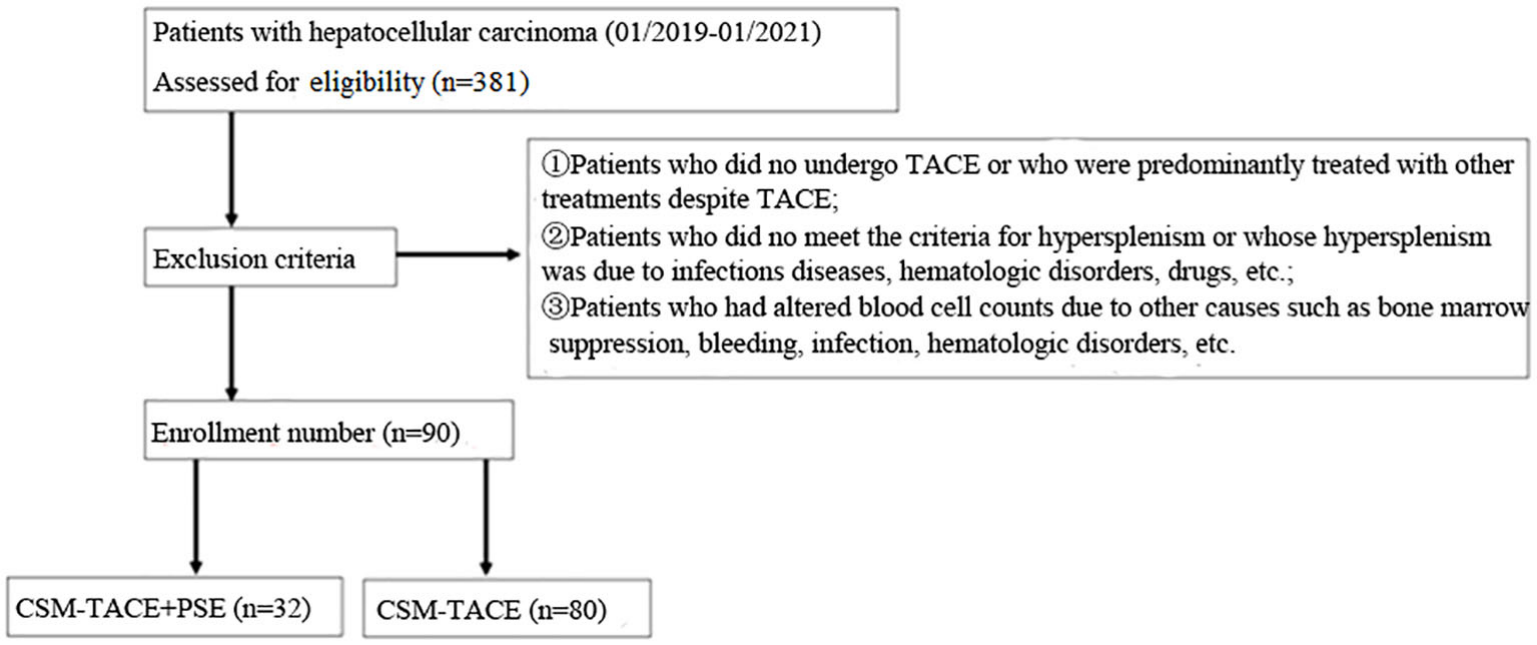
Flow diagram shows the selection of hepatocellular carcinoma patients.

Inclusion criterias: (1) Patients who clinically or pathologically confirmed as HCC; (2) Patients with BCLC stage B-C and Child class A or B; (3) Patients who meet the diagnostic criteria for hypersplenism; (4) Patients who did not receive surgical resection, radiofrequency ablation and other surgical treatment methods before CSM-TACE; (5) Patients without other anti-tumor treatment such as immunotherapy, targeted drugs and traditional Chinese medicine; (6) Patients have liver tumors accounted for less than 70% of the liver volume; (7) Patients without main portal vein tumor thrombus and extensive distant metastasis; (8) Patients were informed that they would receive CSM-TACE combined with PSE or CSM-TACE alone before surgery, and were enrolled voluntarily and signed the informed consent form (**Figure 1**).

Exclusion criterias: (1) Patients who received other anti-tumor therapy before surgery, or refused CSM-TACE treatment; (2) Patients with tumors occupying ≥70% of the entire liver; (3) Patients with ECOG physical strength score > 2 points, or Child grade C; (4) Patients who suffer from infectious diseases, blood system diseases (including primary thrombocytosis, idiopathic thrombocytopenia, anemia of various causes, etc.) and various diseases that require antiplatelet drugs.

### 2.2 Methods

#### 2.2.1 Standardized CSM-TACE

Epirubicin 60mg was applied and mixed with 300-500um CalliSpheres microspheres 1.0g for 30 minutes. Arteriography was performed to find all the blood supply arteries to the liver tumors, and microcatheters were applied to hyper-select the target blood vessels. Slowly and uniformly inject the CalliSpheres microspheres alternately with the contrast agent until the tumor staining disappears completely, and if necessary, gelatin sponge particles can be used for supplementary embolization. The criteria of the end of embolization criteria: stagnation of blood flow in the target vessel or complete disappearance of tumor staining in the contrast display.

#### 2.2.2 Standardized PSE

Apply 500~700μm 8spheres^®^ beads (product registration number: National Machinery Labeling 20153131072, size: 1.0g; Suzhou Hengrui Jia Lisheng Biomedical Technology Co., Ltd., China) mixed with gentamicin 80,000 U and normal saline Embolize.

The catheter was inserted into the end of the splenic artery trunk to further determine the extent of staining of the entire spleen and to compare with preoperative CT or MRI imaging and biochemical indicators. Embolization was performed by mixing 500-700 μm 8spheres^®^ beads (Product Registration No.: National Machinery Labeling 20153131072, Specification: 1.0g; Suzhou Hengrui Callisyn Biomedical Technology Co., China) with gentamicin 80,000 U and normal saline. The catheter was placed into the splenic artery bifurcation, and the embolic agent enters the branch artery with the dominant flow of the splenic artery. The degree of embolization depends mainly on the degree of hypersplenism, taking into account the splenic blood flow rate and the patient’s liver function. Interventional embolization endpoint: the volume of splenic embolization confirmed by imaging did not exceed 50% of the total volume of the spleen. Oral enteric probiotics were started 3 days before surgery, and antibiotics were applied 1 day before surgery and 3 consecutive days after surgery.

#### 2.2.3 Efficacy evaluation

(1) Evaluation of laboratory examination: Peripheral blood monitoring was performed before and 1 month after surgery, including blood routine: white blood cells (WBC), platelet (PLT) count, red blood cell (RBC) count; liver function: albumin (ALB); alanine aminotransferase (ALT), aspartate aminotransferase (AST), serum total bilirubin (TBIL), and cholinesterase (CHE).
(2) Imaging evaluation: abdominal imaging examinations were performed before surgery, 4 days after surgery and 1 month after surgery to observe the blood supply characteristics of HCC, the degree of postoperative necrosis and residual activity, and the scope, volume and morphology of spleen infarction after PSE. At the same time, paid attention to the occurrence of pleural effusion, splenic abscess and portal vein thrombosis.

#### 2.2.4 Survival time and prognostic factors

The survival rates and survival times of patients after the intervention were observed and summarized. Patients who refused re-interventional therapy or underwent other comprehensive treatment after surgery were given continued clinical follow-up and observation, and the follow-up cut-off criteria were patient death or end of follow-up. A multifactorial analysis of prognosis was performed after the follow-up cut-off to investigate the factors affecting prognosis.

Observation and management of complications

The incidence of postoperative adverse reactions and complications was recorded and evaluated, common complications include embolic syndrome, fever, nausea and vomiting, and abdominal pain, as well as serious adverse reactions, including acute liver failure, acute cholecystitis, upper gastrointestinal bleeding, liver abscess, and pulmonary embolism, and the complications of patients in Group include splenic abscess, pancreatitis, and acute thrombotic events. Aggressive comprehensive symptomatic treatment such as analgesia and anti-inflammation was given according to the complications.

### 2.3 Statistical analysis

The statistical software SPSS 20.0 was used for data processing and analysis, and the data conforming to the normal distribution were expressed as mean ± SD. The measurement data between groups was analyzed and compared by independent T-test, and the count data were analyzed by chi-square test or Fisher’s exact test, Kaplan-Meier was used for survival analysis, and log-rank tests were used to compare survival rate. p < 0.05 indicating a statistically significant difference.

## 3 Results

### 3.1 Chronological changes in peripheral blood cell counts

In the CSM-TACE+PSE group, WBC and PLT in peripheral blood of patients after surgery were higher than those before surgery, and the differences were statistically significant (P<0.05); there was no significant difference in the RBC in peripheral blood before and after surgery (P > 0.05) (**Table 2**). In the CSM-TACE group, the WBC decreased at 1 week and 1 month after surgery, and then increased again, and the WBC at 3 months after surgery was still lower than that before surgery, and the difference was statistically (P<0.05). Postoperative peripheral blood RBC was decreased and was lower than that before surgery, and there was statistically significant difference in RBC level between 1 month after surgery and before surgery (P= 0.044) (**Tables 2**–**4**).

**Table 2:** Follow-up results of red blood cells (RBC) counts (×10^12^/L).

Time	CSM-TACE+PSE		CSM-TACE		P2
	Mean ± SD	P1	Mean ± SD	P1
Pre-treatment	3.03 ± 0.60		3.07 ± 0.51		0.369
Post-treatment					
1 wk	2.95 ± 0.83	0.330	2.94 ± 0.57	0.099	0.473
1 mon	2.92 ± 0.47	0.209	2.91 ± 0.49	0.044	0.463
3 mon	3.15 ± 0.60	0.213	2.98 ± 0.60	0.193	0.100

P1: Comparison of RBC counts before and after treatment at different time points within each group; P2: Comparison of RBC counts between the two groups at different time points.

**Table 3:** Follow-up results of white blood cells (WBC) counts (×10^9^/L).

Time	CSM-TACE+PSE		CSM-TACE		P2
	Mean ± SD	P1	Mean ± SD	P1
Pre-treatment	2.41 ± 0.45		2.40 ± 0.50		0.463
Post-treatment					
1 wk	7.62 ± 0.96	<0.001	1.88 ± 0.49	<0.001	<0.001
1 mon	6.33 ± 0.78	<0.001	1.83 ± 0.76	<0.001	<0.001
3 mon	5.06 ± 0.46	<0.001	2.13 ± 0.59	0.004	<0.001

P1: Comparison of WBC counts before and after treatment at different time points within each group; P2: Comparison of WBC counts between the two groups at different time points.

**Table 4:** Follow-up results of platelet (PLT) counts (×10^12^/L).

Time	CSM-TACE+PSE		CSM-TACE		P2
	Mean ± SD	P1	Mean ± SD	P1
Pre-treatment	46.96 ± 9.49		45.62 ± 9.44		0.261
Post-treatment					
1 wk	170.21 ± 26.43	<0.001	30.56 ± 7.51	<0.001	<0.001
1 mon	125.76 ± 18.53	<0.001	41.46 ± 6.29	0.003	<0.001
3 mon	111.98 ± 13.43	<0.001	39.15 ± 10.90	0.0004	<0.001

P1: Comparison of PLT counts before and after treatment at different time points within each group; P2: Comparison of PLT counts between the two groups at different time points.

### 3.2 Hepatic function changes in peripheral blood cell counts

ALT and AST increased transiently at 1week after surgery in both groups, and there were statistically significant differences in ALT and AST in the CSM-TACE group compared with those before surgery (P<0.05). There were no significant differences in ALT and AST at 1 and 3 months after surgery compared with those before surgery (P>0.05). The postoperative CHE in the CSM-TACE+PSE group increased gradually, while the CHE in the CSM-TACE group decreased temporarily and then recovered to the preoperative level, and there was a statistical difference before surgery and 1 week after surgery (P < 0.05), there were statistical differences between the two groups at different time points (**Tables 5**–**9**).

**Table 5:** Follow-up results of alanine aminotransferase (ALT) counts (U/L).

Time	CSM-TACE+PSE		CSM-TACE		P2
	Mean ± SD	P1	Mean ± SD	P1
Pre-treatment	53.71 ± 25.19		56.38 ± 15.94		0.270
Post-treatment					
1 wk	60.75 ± 33.03	0.171	87.28 ± 17.93	<0.001	<0.001
1 mon	46.03 ± 15.20	0.072	50.82 ± 21.03	0.056	0.129
3 mon	45.40 ± 14.67	0.056	52.95 ± 17.73	0.138	0.137

P1: Comparison of ALT counts before and after treatment at different time points within each group; P2: Comparison of ALT counts between the two groups at different time points.

**Table 6:** Follow-up results of aspartate aminotransferase (AST) counts (U/L).

Time	CSM-TACE+PSE		CSM-TACE		P2
	Mean ± SD	P1	Mean ± SD	P1
Pre-treatment	56.06 ± 24.15		53.37 ± 17.25		0.271
Post-treatment					
1 wk	61.46 ± 40.64	0.260	67.49 ± 21.53	<0.001	0.179
1 mon	49.59 ± 20.27	0.125	48.27 ± 18.38	0.063	0.377
3 mon	51.81 ± 21.33	0.229	50.49 ± 20.60	0.208	0.387

P1: Comparison of AST counts before and after treatment at different time points within each group; P2: Comparison of AST counts between the two groups at different time points.

**Table 7:** Follow-up results of albumin (ALB) counts (g/L).

Time	CSM-TACE+PSE		CSM-TACE		P2
	Mean ± SD	P1	Mean ± SD	P1
Pre-treatment	38.53 ± 5.99		40.18 ± 5.46		0.094
Post-treatment					
1 wk	36.65 ± 5.02	0.089	38.25 ± 9.22	0.086	0.183
1 mon	37.09 ± 5.81	0.166	39.92 ± 11.58	0.438	0.099
3 mon	40.12 ± 8.84	0.201	41.31 ± 4.77	0.119	0.204

P1: Comparison of ALB counts before and after treatment at different time points within each group; P2: Comparison of ALB counts between the two groups at different time points.

**Table 8:** Follow-up results of serum total bilirubin (TBIL) counts (mmol/L).

Time	CSM-TACE+PSE		CSM-TACE		P2
	Mean ± SD	P1	Mean ± SD	P1	
Pre-treatment	21.83 ± 7.97		20.47 ± 4.53		0.152
Post-treatment					
1 wk	24.91 ± 10.40	0.094	21.78 ± 6.71	0.110	0.053
1 mon	20.63 ± 7.46	0.268	19.34 ± 2.76	0.054	0.120
3 mon	22.28 ± 4.70	0.189	20.59 ± 4.95	0.446	0.059

P1: Comparison of TBIL counts before and after treatment at different time points within each group; P2: Comparison of TBIL counts between the two groups at different time points.

**Table 9:** Follow-up results of cholinesterase (CHE) counts (U/L).

Time	CSM-TACE+PSE		CSM-TACE		P2
	Mean ± SD	P1	Mean ± SD	P1
Pre-treatment	5006.19 ± 1925.58		5658.80 ± 1746.27		0.053
Post-treatment					
1 wk	5278.83 ± 2217.36	0.301	3892.58 ± 1886.82	<0.001	0.001
1 mon	6546.89 ± 2068.75	0.001	5473.80 ± 2383.41	0.317	0.018
3 mon	6357.54 ± 1908.18	0.003	5535.80 ± 1623.15	0.346	0.017

P1: Comparison of CHE counts before and after treatment at different time points within each group; P2: Comparison of CHE counts between the two groups at different time points.

### 3.3 Post-interventional imaging evaluation

**Figure 2:**
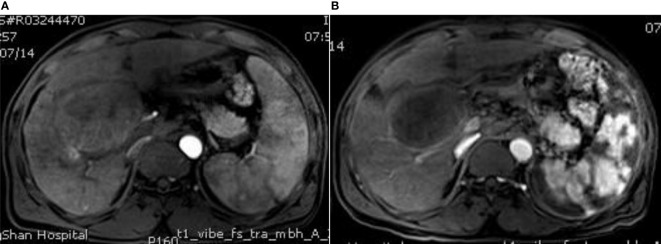
MRI image before surgery and MRI image after surgery. **(A)** The contrast-enhanced MRI of the liver shows multiple space-occupying lesions, the larger lesions are located in the right lobe of the liver, which adjacent to the hepatic hilum, moderate enhancement and splenic enlargement can be observed in the arterial phase; **(B)** Enhanced MRI of the upper abdomen at 3 months after surgery showes that the liver tumor shrank without obvious enhancement, the spleen shows piebald necrosis, the spleen infarct area accounts for about 50%, and the platelets and leukocytes increase to the normal level and are stable.

**Figure 3:**
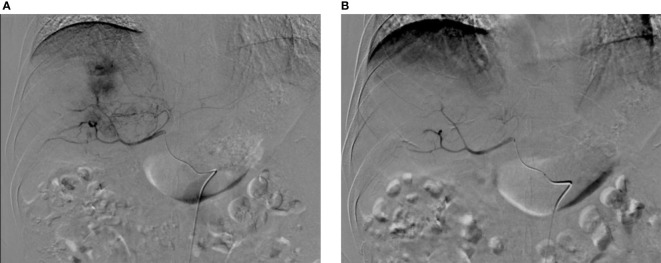
Digital subtraction angiography (DSA) before and after surgery. **(A)** DSA before CSM-TACE shows intrahepatic mass tumor staining, and the feeding arteries mainly come from the right hepatic artery; **(B)** After embolization with CSM particles, the target blood vessels were blocked, and postoperative DSA shows the tumor staining disappeared.

**Figure 4:**
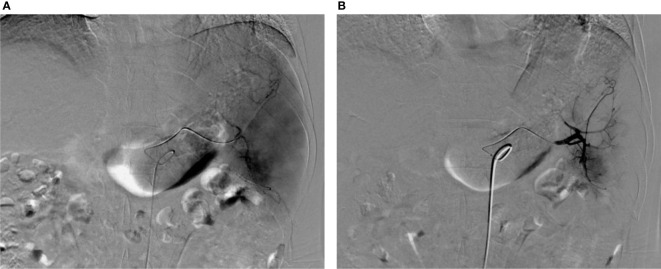
Images before and after PSE. **(A)** Splenic arteriography before PSE showed thickening and tortuous splenic artery and enlarged spleen staining; **(B)** Angiography after PSE shows that the peripheral staining of the spleen disappeared, and the embolization volume is about 50%.

According to the mRECIST evaluation criteria, 1 month after the first intervention in both groups, the objective response of liver tumor in the experimental group was complete response (CR) in 13 (40.63%) patients, partial response (PR) in 17 (53.13%) patients, stable disease (SD) in 2 (6.25%) patients, and progression disease in 0 (0%) patients, with an objective response rate (ORR) of 93.75%. The objective response of liver tumor in control group was CR in 25 (44.83%) patients, PR in 29 (48.28%) patients, SD in 3 (5.17%) patients, and PD in 1 (1.72%) patient, with an ORR of 93.10%. There was no significant difference in ORR between the two groups 1 month after interventional surgery (P=0.455) (**Figures 2**–**4**).

### 3.4 Survival time

**Figure 5:**
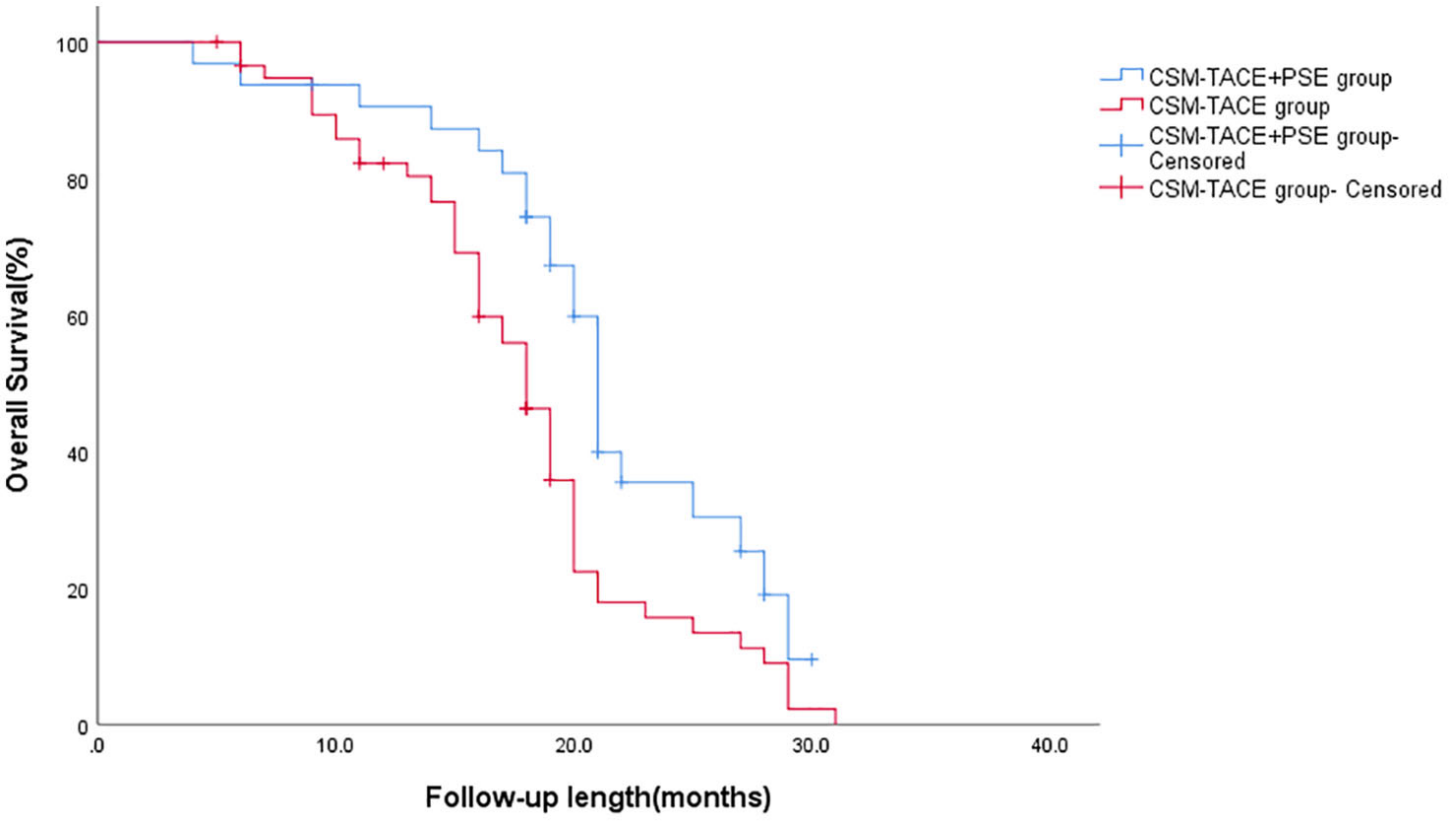
The overall survival time.

The follow-up ended in June 2022, and no patients were lost to follow-up. The follow-up time was 4-31 months, on average (17.84 ± 6.27) months. The 12-month survival rate in the CSM-TACE+PSE and CSM-TACE groups was 95% and 80.3%, respectively, and the 24-month survival rate in the CSM-TACE+PSE and the CSM-TACE groups were 35.4% and 15.6%, respectively. The survival rate of the CSM-TACE+PSE group was compared with that of the CSM-TACE group, 12-month survival rate: of 90.5% VS 80.3% (P=0.033), 24-month survival rate: 35.4% VS 15.6% ((P=0.018). mOS: 21 months (95%CI: 20.07-21.93) VS 19 months (95%CI: 17.94-20.06), P=0.006. The 21-month and 19-month median OS were (95% CI:20.07-21.93) and (95% CI:17.94 -20.06), P= 0.006 (**Table 10** and **Figure 5**).

**Table 10:** Prognosis of two group.

Items	CSM-TACE+PSE	CSM-TACE	P
OS	21 (95%CI:20.07-21.93)	19 (95%CI:17.94-20.06)	0.006
Intrahepatic *in situ* progression	9.64 ± 2.19	10.53 ± 3.34	0.065
Intrahepatic neoplastic metastasis	12.41 ± 4.05	10.28 ± 2.30	0.004
Extrahepatic progression	17.23 ± 4.95	14.78 ± 4.08	0.006

### 3.5 Prognostic factors

A total of 9 indicators including age, gender, liver function grade, tumor size, type of hypersplenism and whether combined with PSE were included. The Cox model was used for multivariate regression analysis on the prognostic factors in HCC with hypersplenism. The results showed that non-PSE/PSE and hypersplenism were prognostic factors, among which, the degree of hypersplenism was a risk factor affecting prognosis, and PSE was a protective factor affecting prognosis, hazard ratio (HR): 0.322 (95%CI: 0.155-0.668) (**Table 11**).

**Table 11:** Univariate and multivariate analysis on parameters affecting overall survival (OS).

Parameter	Univariable analysis			Multivariable analysis		
	HR	95% CI	P	HR	95% CI	P
N-PSE/PSE	0.453	0.220-0.936	0.032	0.322	0.155-0.668	0.002
Gender (Male/Female)	2.964	0.397-9.148	0.290	1.648	0.212-12.891	0.600
Age (Elderly/middle-aged)	0.979	0.356-1.563	0.085	0.968	0.178-1.663	0.057
Hepatitis (Y/N)	0.760	0.409-1.412	0.385	0.811	0.488-1.467	0.488
Child-Pugh (A/B)	2.507	0.867-7.193	0.055	2.403	0.602-8.223	0.171
BCLC(B/C)	1.467	0.901-3.543	0.101	0.336	0.325-1.123	0.604
Diameter (≤5cm/>5cm)	1.351	0.871-2.192	0.734	1.135	0.672-1.921	0.811
Tumor number (Solitary/Multiple)	1.59	1.02-2.49	0.031	1.21	0.93-1.56	0.175
PVTT (Y/N)	0.722	0.579-2.176	0.733	0.994	0.524-1.883	0.984
Hypersplenism (mild to moderate/severe)	4.330	1.257-14.809	0.021	4.588	1.344-15.655	0.014

### 3.6 Comparison of related complications between the two groups

Patients in both groups were routinely treated with hepatoprotection, acid suppression, gastric mucosa protection and fluid replacement therapy after surgery. For patients with large intrahepatic tumors or PSE, antibiotics were intensified and gastrointestinal flora was regulated, and enemas should be given if necessary to maintain intestinal patency. The main postoperative adverse reactions in two groups included fever, nausea and vomiting, and abdominal pain, although the results showed that the incidence rate of adverse reactions was higher in the CSMs-TACE group, there was no statistical difference in the incidence rate between the two groups. There were no significant differences in pleural effusion and ascites between two groups. In CSMs-TACE+PSE group, 1 patient had portal thrombosis, which completely disappeared after comprehensive treatment, with an incidence rate of 3.13% (1/32). No other serious complications such as splenic abscess was obversed in CSMs-TACE+PSE group (**Table 12**).

**Table 12:** Complications observed in 90 patients at 1 week after surgery.

Complications	CSM-TACE+PSE [n (%)]		CSM-TACE		P1	P2
	Any gradeNumber [n (%)]	≥grade 3Number[n (%)]	Any gradeNumber [n (%)]	≥grade 3Number [n (%)]		
Abdominal pain	13 (40.63)	0	23 (39.66)	0	0.119	–
Fever	10 (31.25)	0	17 (29.31)	0	0.846	–
Nausea and vomiting	20 (62.50)	3 (9.38)	36 (62.07)	5 (8.62)	0.968	0.904
Pleural effusion or ascites	6 (18.75)	0	10 (17.24)	0	0.857	–
Abdominal distention	12 (37.50)	0	17 (29.31)	0	0.426	–
Constipation	3 (9.37)	0	6 (10.34)	0	0.883	–
Portal vein thrombosis	1 (3.13)	0	0	0		–
Bacterial peritonitis	1 (3.13)	0	2 (3.45)	0	0.946	–

P1: Comparison of total adverse events between two groups; P2: Comparison of grade ≥3 adverse events between two groups.

## 4 Discussions

We believe several factors contributed to the good outcome of PSE in this study. First factor is the choice of embolization particles. For a long time in the past, homemade gelatin sponges have been used as embolic agents in PSE. Permanent embolic materials achieved better laboratory and radiological outcomes than gelatin sponge particles in PSE of cirrhotic hypersplenism patients (13, 14).

Zhu K et al. believe that PSE using polyvinyl alcohol (PVA) particles can achieve even better efficacy in alleviating hypersplenism. Although, Tetsuya MasadaK, et al. think that the efficacy of PSE is similar to the use of coils versus gelatin particles (15). Iodine oil, a traditional type of embolic agent used in TACE surgery, has also been rarely reported for PSE. Romaric Loffroy et al. showed that PSE with Glubran ^®^ 2/Lipiodol ^®^ mixture is safe and effective in the management of thrombocytopenia related to hypersplenism in cancer patients. It allows sufficient platelet count improvement for administration of systemic chemotherapy (16).

In this study, the embolic agent 8spheres^®^ was used in PSE, which was independently developed in China. As a new domestic PVA particle, 8spheres^®^ has better histocompatibility, which can not only permanently embolize blood vessels, but also significantly reduce inflammatory response, and in clinical studies, it shows a lower incidence rate of fever and pain. However, Mohamed M A Zaitoun et al. showed that permanent particles were associated with greater abdominal pain, this was not completely consistent with the results in this study (13).

The second is the strict control of the embolization area during PSE. In the field of PSE, the embolization area is an important factor of general concern. PSE can reduce the platelet pool and induce an increase in platelet count. This increase is greatly dependent on the infarcted splenic volume [5, 14, 17–21]. Clinical studies have concluded that controlling the splenic embolism at 50%-70% can ensure efficacy and reduce the related complications (14). Mingyue Cai, et al. found that a large infarcted splenic volume and a high Child-Pugh score may cause complications (20). In this study, we controlled the embolization area to 50%, and our cases were HCC patients with hypersplenism, and Child-Pugh class C patients were excluded. The results of this study also confirmed that a one-time control of the embolization area to 50% can achieve good short-term efficacy. In addition, this study also suggested that 8spheres^®^ of 500-700 μm may be more suitable for the clinical application of PSE (22), and it is economical and controllable, thereby further reducing the occurrence of complications.

In addition to the good efficacy in increasing white blood cells and platelets, this study has the following two obvious advantages. First of all, in terms of clinical study design, we used domestic drug-loaded microspheres CalliSpheres^®^ for CSM-TACE, and domestic 8spheres^®^ was used for PSE. Such a simultaneous treatment combination is an innovative point of this study, which not only solves two medical problems at the same time, but also shows good tolerance in patients, reduces medical costs and avoids repetitive medical waste. Ishikawa T, et al. reported that the application of lipiodol-TACE simultaneous splenic embolization in the treatment of HCC with hypersplenism not only had good safety and clinical efficacy, but also significantly improved the Child-Pugh score after surgery (9). In addition, Hidaka H et al. reported a clinical study of ablation therapy for HCC after PSE, and they think that PSE is a good supplementary method for the treatment of HCC (4). So whether it is simultaneous or metachronous combination, there is a consensus on the value of PSE in the treatment of hepatocellular carcinoma combined with cirrhosis.

Our preliminary clinical studies have confirmed the feasibility of drug-loaded microspheres in the treatment of unresectable large HCC, and several studies in recent years have also confirmed that DEB-TACE has a better anti-tumor effect and less adverse effects than c-TACE, and has gradually become the preferred method for the treatment of HCC. However, the application of DEB-TACE in combination with PSE for HCC combined with hypersplenism has not been reported, and the results of this study showed that the combination of DEB-TACE with PSE was well tolerated by the patients, and the incidence of complications was not significantly increased compared with the control group, indicating that DEB-TACE combined with PSE for the treatment of HCC combined with hypersplenism is completely feasible. Domestic 8spheres^®^ is a new type of domestic PVA particles, and it has been reported that PSE with 8spheres^®^ has better safety and good efficacy (22). Although the results of this study showed that the ORR was above 90% in both groups at 1 month after surgery, also suggested that CSM-TACE simultaneous PSE could improve the median OS of patients with HCC and hypersplenism, and the median OS in the observation group was 2 months loner than that in the control group, which is basically consistent with the (24.47 ± 3.68) months reported by Liu et al. (10). However, the sample size in our study is significantly larger.

Secondly, hemodynamics caused by CSM-TACE+PSE improved the liver functional reserve of patients, which was also confirmed in this study. While, the hemodynamic changes are the main cause of hypersplenism caused by liver cirrhosis, and the formation of hypersplenism further reduces blood flow back to the liver in patients with cirrhosis (21), which is an important adverse factor affecting the prognosis of HCC. Tsuyoshi Ishikawa et al. concluded that simultaneous TACE+PSE was beneficial for patients’ liver functional reserve, and their recent study also showed that PSE is expected to produce hepatic arterial buffer response (HABR)-mediated hepatic functional improvements in cirrhosis patients with Child-Pugh class B/C (9, 23). Our study also confirmed that simultaneous CSM-TACE and PSE using 8spheres^®^ can increase the level of cholinesterase, suggesting that CSM-TACE+PSE can improve liver functional reserve capacity by improving hemodynamics (24), which is particularly important for the prognosis of patients with advanced HCC and therefore an important factor in achieving a good prognosis.

The main adverse reactions in this study were post-embolization syndrome, including varying degrees of fever, nausea, vomiting, and epigastric pain (22, 25, 26). After symptomatic treatment, these symptoms gradually relieved and disappeared in a short period of time, although patients in experimental group received CSM-TACE+PSE, and there was no significant difference in the degree and incidence of abdominal pain between the two groups. Splenic abscess, acute pancreatitis, and spleen-portal vein thrombosis are serious complications of PSE, with an incidence of about 10% (27–29). In terms of spleen-portal vein thrombosis, the incidence of surgical splenectomy is higher than that of PSE, A systematic review and Meta-analysis showed that the incidence rates of the two were 24.6% and 11.7%, respectively (30). The incidence of splenic-portal vein thrombosis in surgical splenectomy is higher than that in PSE, and a systematic review and Meta-analysis showed that the incidence in surgical splenectomy and PSE was 24.6% and 11.7%, respectively (30).

Compared with surgical resection, PSE has a lower incidence of serious complications such as severe infection and thrombosis (28, 30). The incidence of related complications after PSE in this group was extremely low, and the incidence of thrombosis was 3.13%. Application of gentamicin to adequately soak the embolic agent, preoperative intestinal preparation and postoperative prophylactic application of antibiotics, and postoperative oral administration of intestinal probiotics to keep the bowels open during interventional surgery are also important preventive factors, in our opinion. Active fluid replacement should be performed 12-24 hours after surgery to prevent insufficient body fluids, thereby preventing or reducing the occurrence of acute thrombotic events.

In conclusion, CSM-TACE simultaneous PSE can improve the degree of hypersplenism in patients with HCC and liver cirrhosis while effectively controlling intrahepatic tumor lesions. Such a combination treatments not only have an ideal tumor response rate, but also improve and enhance the functional reserve capacity of the liver, and further increasing the median OS. CSM-TACE+PSE provides a good choice for the treatment of advanced HCC patients with hypersplenism. The limitations of this study are the small sample size, selective bias, and short follow-up time. A prospective clinical study design with a large sample is needed to further evaluate the effectiveness and safety of the combination treatment.

## Data availability statement

The original contributions presented in the study are included in the article/supplementary material. Further inquiries can be directed to the corresponding authors.

## Ethics statement

The studies involving human participants were reviewed and approved by the Ethics Committee of Affiliated Zhongshan Hospital of Dalian University.The patients/participants provided their written informed consent to participate in this study.

## Author contributions

JZ and ZF: Responsible for clinical trial research and paper writing. SL, XL and YL: Responsible for patient follow-up and data statistics. FG, JS and YZ: Responsible for experiment management. GZ and MZ: Responsible for project design and experimental implementation. All authors contributed to the article and approved the submitted version.

## Conflict of interest

The authors declare that the research was conducted in the absence of any commercial or financial relationships that could be construed as a potential conflict of interest.

## Publisher’s note

All claims expressed in this article are solely those of the authors and do not necessarily represent those of their affiliated organizations, or those of the publisher, the editors and the reviewers. Any product that may be evaluated in this article, or claim that may be made by its manufacturer, is not guaranteed or endorsed by the publisher.

## References

[1] Mu XM Wang W Jiang YY Feng J. Patterns of comorbidity in hepatocellular carcinoma: A network perspective. Int J Environ Res Public Health (2020) 17(9):3108. doi: 10.3390/ijerph17093108 32365650 PMC7246663

[2] Jia Z Zhang K Jiang L Huang RH He R Wang ZJ. Simultaneous radiofrequency ablation combined with laparoscopic splenectomy: a safe and effective way for patients with hepatocellular carcinoma complicated with cirrhosis and hypersplenism. Minim Invasive Ther Allied Technol (2020) 29(3):177–84. doi: 10.1080/13645706.2019.1609990 31116622

[3] Zhang XY Li C Wen TF Peng W Yan LN Li B. Synchronous splenectomy and hepatectomy for patients with small hepatocellular carcinoma and pathological spleen: neutrophil to lymphocyte ratio changes can predict the prognosis. Oncotarget (2017) 8(28):46298–311. doi: 10.18632/oncotarget.17758 PMC5542268 28549349

[4] Hidaka H Kokubu S Nakazawa T Minamino T Takada J Tanaka Y. Therapeutic benefits of partial splenic embolization for thrombocytopenia in hepatocellular carcinoma patients treated with radiofrequency ablation. Hepatol Res (2009) 39:772–8. doi: 10.1111/j.1872-034X.2009.00508.x 19473438

[5] Amin MA El Gendy MM Dawoud IE Shoma A Negm AM Amer TA. Partial splenic embolization versus splenectomy for the management of hypersplenism in cirrhotic patients. World J Surg (2009) 33:1702–10. doi: 10.1007/s00268-009-0095-2 19513783

[6] Jiang WT Yang J Xie Y Guo QJ Tian DZ Li JJ. Simultaneous partial splenectomy during liver transplantation for advanced cirrhosis patients combined with severe splenomegaly and hypersplenism. World J Gastroenterol (2021) 27(7):654–65. doi: 10.3748/wjg.v27.i7.654 PMC7901050 33642835

[7] Hirooka M Koizumi Y Tanaka T Nakamura Y Sunago K Yukimoto A. Treatment on the spleen prevents the progression of secondary sarcopenia in patients with liver cirrhosis. Hepatol Commun (2020) 4(12):1812–23. doi: 10.1002/hep4.1604 PMC7706300 33305152

[8] Rong JJ Liu D Liang M Wang QH Sun YJ Quan YZ. The impacts of different embolization techniques on splenic artery embolization for blunt splenic injury: a systematic review and meta-analysis. Mil Med Res (2017) 4:17. doi: 10.1186/s40779-017-0125-6 28573044 PMC5450228

[9] Ishikawa T Kubota T Horigome R Kimura N Honda H Iwanaga A. Concurrent partial splenic embolization with transcatheter arterial chemoe mbolization for hepatocellular carcinoma can maintain hepatic functional reserve. Hepatol Res (2014) 44:1056–61. doi: 10.1111/hepr.12222 23941627

[10] Liu JB Wu ZJ Zhang JX Xie YF Sun P Wu HY. Effect of partial splenic embolization on transarterial chemoembolization for hepatocellular carcinoma with hypersplenism. Med (Baltimore) (2021) 100(26):e26441. doi: 10.1097/MD.0000000000026441 PMC8257862 34190168

[11] Zhao GS Liu S Zhang YW Zhao T Wang RY Bian J. Irinotecan eluting beads-transarterial chemoembolization using callispheres® microspheres is an effective and safe approach in treating unresectable colorectal cancer liver metastases. Ir J Med Sci (2022) 191(3):1139–45. doi: 10.1007/s11845-021-02629-9 PMC9135896 34264426

[12] Zhao GS Liu S Chen SB Ren ZZ Li C Bian J. Assessment of efficacy and safety by CalliSpheres versus HepaSpheres for drug-eluting bead transarterial chemoembolization in unresectable large hepatocellular carcinoma patients. Drug Delivery (2021) 28(1):1356–62. doi: 10.1080/10717544.2021.1943057 PMC8245102 34180755

[13] Zaitoun MMA Alkhalik Basha MA Elsayed SB El Deen DS Zaitoun NA Alturkistani H. Comparison of three embolic materials at partial splenic artery embolization for hypersplenism: Clinical, laboratory, and radiological outcomes. Insights Imaging (2021) 12(1):85. doi: 10.1186/s13244-021-01030-5 34173891 PMC8236018

[14] Zhu KS Meng XC Li ZR Huang MS Guan SH Jiang ZB. Partial splenic embolization using polyvinyl alcohol particles for hypersplenism in cirrhosis: A prospective randomized study. Eur J Radiol (2008) 66:100–6. doi: 10.1016/j.ejrad.2007.04.010 17532166

[15] Masada T Tanaka T Sakaguchi H Nakagomi M Miura Y Hidaka T. Coils versus gelatin particles with or without intraarterial antibiotics for partial splenic embolization: A comparative evaluation. J Vasc Interv Radiol (2014) 25(6):852–8. doi: 10.1016/j.jvir.2013.12.563 24534093

[16] Loffroy R Falvo N Nakaï M Pescatori L Aho-Gléglé S Gehin S. Partial splenic embolization with glubran ® 2/Lipiodol ® mixture for oncological patients with hypersplenism-related thrombocytopenia requiring systemic chemotherapy. Quant Imaging Med Surg (2019) 9(3):409–17. doi: 10.21037/qims.2019.03.07 PMC6462571 31032188

[17] Hayashi H Beppu T Masuda T Mizumoto T Takahashi M Ishiko T. Predictive factors for platelet increase after partial splenic embolization in liver cirrhosis patients. J Gastroenterol Hepatol (2007) 22:1638–42. doi: 10.1111/j.1440-1746.2007.05090.x 17683504

[18] Zhu K Meng X Qian J Huang M Guan S Jiang Z. Partial splenic embolization for hypersplenism in cirrhosis: A long-term outcome in 62 patients. Dig Liver Dis (2009) 41(6):411–6. doi: 10.1016/j.dld.2008.10.005 19070555

[19] Wu BG Chou ASB Hoo GJ Lee MC. Eighty percent partial splenic embolization is a safe and effective procedure in management of cirrhotic hypersplenism. Formos J Surg (2017) 50:101. doi: 10.4103/fjs.fjs_35_17

[20] Cai MY Huang WS Lin CS Li ZR Qian JS Huang MS. Partial splenic embolization for thrombocytopenia in liver cirrhosis: Predictive factors for platelet increment and risk factors for major complications. Eur Radiol (2016) 26(2):370–80. doi: 10.1007/s00330-015-3839-4 26002134

[21] Talakić E Schaffellner S Kniepeiss D Mueller H Stauber R Quehenberger F. CT perfusion imaging of the liver and the spleen in patients with cirrhosis: Is there a correlation between perfusion and portal venous hypertension? Eur Radiol (2017) 27(10):4173–80. doi: 10.1007/s00330-017-4788-x PMC5579174 28321540

[22] Lu HH Zheng CS Liang B Xiong B. Quantitative splenic embolization possible: Application of 8Spheres conformal microspheres in partial splenic embolization (PSE). BMC Gastroenterol (2021) 21(1):407. doi: 10.1186/s12876-021-01991-3 34706678 PMC8555096

[23] Ishikawa T Sasaki R Nishimura T. Short-term effects of hepatic arterial buffer responses induced by partial splenic embolization on the hepatic function of patients with cirrhosis according to the child-pugh classification. Intern Med (2021) 60(9):1331–42. doi: 10.2169/internalmedicine.6267-20 PMC8170249 33281164

[24] Helaly AZ Al-Warraky MS El-Azab GI. Portal and splanchnic hemodynamics after partial splenic embolization in cirrhotic patients with hypersplenism. APMIS (2015) 123(12):1032–9. doi: 10.1111/apm.12470 26547369

[25] Sakai T Shiraki K Inoue H Sugimoto K OhmoriK S Murata K. Complications of partial splenic embolization in cirrhotic patients. Dig Dis Sci (2002) 47:388–91. doi: 10.1023/A:1013786509418 11855556

[26] N'Kontchou G Seror O Bourcier V Mohand D Ajavon Y Castera L. Partial splenic embolization in patients with cirrhosis: efficacy, tolerance and long-term outcome in 32 patients. Eur J Gastroenterol Hepatol (2005) 17:179–84. doi: 10.1097/00042737-200502000-00008 15674095

[27] Zhang L Zhang ZG Long X Liu FL Zhang WG. Severe complications after splenic artery embolization for portal hypertension due to hepatic cirrhosis. Risk Manag Healthc Policy (2020) 13:135–40. doi: 10.2147/RMHP.S234628 PMC7037048 32110126

[28] Wu YY Li HY Zhang TS. Splanchnic vein thrombosis in liver cirrhosis after splenectomy or splenic artery embolization: A systematic review and meta-analysis. Adv Ther (2021) 38(4):1904–30. doi: 10.1007/s12325-021-01652-7 33687650

[29] Ogawa S Yamamoto A Jogo A Nakano MM Kageyama K Sohgawa E. Splenic vein diameter is a risk factor for the portal venous system thrombosis after partial splenic artery embolization. Cardiovasc Intervent Radiol (2021) 44(6):921–30. doi: 10.1007/s00270-020-02751-8 PMC8172394 33474605

[30] Aiolfi A Inaba K Strumwasser A. Splenic artery embolization versus splenectomy: Analysis for early in-hospital infectious complications and outcomes. J Trauma Acute Care Surg (2017) 83(3):356–60. doi: 10.1097/TA.0000000000001550 28459796

